# Liver Damage and microRNAs: An Update

**DOI:** 10.3390/cimb45010006

**Published:** 2022-12-23

**Authors:** Erika Cione, Diana Marisol Abrego Guandique, Maria Cristina Caroleo, Filippo Luciani, Manuela Colosimo, Roberto Cannataro

**Affiliations:** 1 Department of Pharmacy, Health and Nutritional Sciences, University of Calabria, 87036 Rende, Italy; 2 Galascreen Laboratories, University of Calabria, 87036 Rende, Italy; 3 Department of Health Sciences, University of Magna Graecia Catanzaro, 88100 Catanzaro, Italy; 4 Infectious Disease Unit Annunziata Hospital, 87100 Cosenza, Italy; 5 Microbiology Unit Pugliese Ciaccio Hospital, 88100 Catanzaro, Italy

**Keywords:** early biomarkers, microRNAs, drug-induced liver injury, hepatitis B virus infection, hepatitis B virus infection

## Abstract

One of the major organs in the body with multiple functions is the liver. It plays a central role in the transformation of macronutrients and clearance of chemicals and drugs. The serum biomarkers often used to indicate liver damage are not specifically for drug-induced liver injury (DILI) or liver injury caused by other xenobiotics, nor for viral infection. In this case, microRNAs (miRNAs) could play an exciting role as biomarkers of specific liver damage. In this review, we aimed to update the current literature on liver damage induced by drugs, as acute conditions and viral infections mediated by the hepatitis B virus (HBV) linked these two conditions to advanced research, with a focus on microRNAs as early biomarkers for liver damage. The undoubtable evidence that circulating miR-122 could be used as a human biomarker of DILI came from several studies in which a strong increase of it was linked with the status of liver function. In infancy, there is the possibility of an early miRNA detection for hepatitis B virus infection, but there are a lack of solid models for studying the HVB molecular mechanism of infection in detail, even if miRNAs do hold unrealized potential as biomarkers for early detection of hepatitis B virus infection mediated by HBV.

## 1. Introduction

The liver is one of the major organs with multiple functions. It plays a central role in transforming macronutrients and clearing chemicals and drugs. The serum biomarkers often used to indicate liver damage are: (i) the transaminases alanine (ALT) and aspartate (AST) that should be three times more of the normal upper limit (NUL) level; (ii) the alkaline phosphatase (ALP), more than double NUL, and (iii) total bilirubin level, more than twice NUL when associated with an increase in both enzymes [1,2]. However, the serum level of these biomarkers is not specifically for drug-induced liver injury (DILI) or liver injury caused by other xenobiotics, nor for viral infection. Therefore, these parameters are unspecific to characterize the type of liver damage. In this scenario, microRNAs (miRNAs) could have an important role as biomarkers of specific liver damage [3]. In this review, we aimed to update the current literature on drug-induced liver damage as an acute condition, viral infections mediated by hepatitis B virus (HBV), and related advances, focusing on microRNAs contributing to liver damage in these two types of conditions.

## 2. Endogenous Liver microRNAs

The liver is responsible for maintaining proper homeostasis of lipid, glycidic, and iron metabolism and xenobiotics detoxification [4]. Since their discovery in 1993, several studies describe the role of miRNAs in liver physiological functions. Additionally, their role in xenobiotic detoxification still needs to be better understood. MiRNAs are single-stranded small non-coding RNAs, between 18 and 22 nucleotides, able to regulate gene expression in various conditions [5,6,7]. The liver miRNome is represented by miR-122 for about 70% [8]. It is worth mentioning that the miR-122 sequence is highly conserved between species, and its expression increases over embryonic liver development and persists throughout adulthood. This miRNA in the hepatocyte interacts with different genes modifying their expression; for example, HNF6, HNFA4, and FOXA1 genes participate in a positive feedback loop with miR-122 to maintain the balance between proliferation and differentiation of the cells during embryonic development [9]. MiR-122 has been the first miRNA related to hepatic lipid metabolism so far. Inhibition of miR-122 expression leads to the downregulation of enzymes involved in lipid metabolism. The inhibition also leads to the upregulation of enzymes participating in β-oxidation. Consequently, a significant reduction in cholesterol associated with a downregulation of genes involved in its synthesis was studied in the murine liver [9]. However, the results in the human hepatocyte cell line (HepG2) are reversed; the silencing of miR-122 leads to an initial increase in HMGCR and SREBP-2, a key protein for cholesterol biosynthesis, and an increase in the AMPK, also significant in lipid metabolism. According to Gatfield et al., suppression of miR-122 hepatic showed a marked decrease in total serum triglyceride levels. This is because it activates MTTP, increasing the concentration of VLDL in the blood [10]. In vivo depletion of miR-122 causes iron deficiency due to increased mRNA expression of the peptide hormone hepcidin which is the key factor of duodenal iron absorption and release [11]. Additionally, the miR-33 is an essential regulator of lipid homeostasis targeting ABCA1, ABCG1, NPC1 [12,13], and key enzymes involved in the oxidation of fatty acids, such as CPT1α, CROT, SIRT6, and AMPKα [13]. Either the miR-23b is involved in lipid metabolism [14], or the miR-1/miR-206 axes can attenuate LXRα-induced lipogenesis by targeting the LXRα mRNA in the hepatocytes [15]. Besides that, it was seen so far that miR-181a downregulation improves hepatic insulin sensitivity [16]. Recently, it was shown that the reactivation of liver miR-192 alleviates steatosis [17]. 

**Figure 1:**
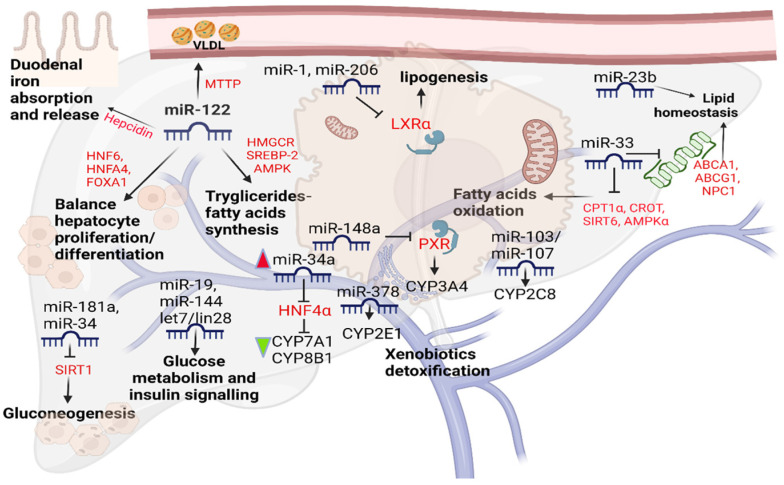
miRNAs maintaining metabolic homeostasis.

The role of miRNAs in the maintaining of metabolic homeostasis it was also studied in glycidic biochemical mechanisms. Hepatic gluconeogenesis is promoted by miR-34a and miR-181a targeting, and therefore inhibits SIRT-1 [16]. Recently, Jia et al. reported in newborn piglets that ssc-miR-339-5p and ssc-miR-532-5p can target the G6PC playing a role in the last steps of glycogenolysis and gluconeogenesis [18]. Similarly, miR-19a activates AKT/GSK and glycogen synthesis by acting on the PTEN expression block [19]. Furthermore, glucose metabolism is affected by miR-144 as regulator of hepatic Krebs cycle in obesity-induced insulin resistance in both mice and humans. MiR-144 drive the fumarase activity preventing NRF2 activation [20]. Additionally, let7/lin28 axis is important in maintaining energetic homeostasis, especially in alterations related to glucose and insulin signaling, in part mediated by repression of insulin-PIK3-mTOR pathways [21]. Despite the loss of mature miRNAs, in Dicer1 knockout in hepatocytes, liver function was maintained, as mirrored by normal blood glucose, cholesterol, bilirubin, and albumin levels, until 2–4 months of age in these mice. After this age, they harbored progressive liver damage and elevated serum of transaminase ALT/AST [4]. Therefore, they do not play an essential role in liver function but in the development of liver damage. In fact, overexpression of miR-122 promotes hepatic differentiation and maturation [22]. Lastly, the liver is the main organ for detoxifying xenobiotics through cytochromes regulation [23]. The cytochrome (CYP) 3A4 is responsible for the greater oxidative degradation of clinically used drugs, and it is regulated by Pregnane X Receptor (PXR), which in turn is inhibited by miR-148a [24]. Other studies reported human liver direct or indirect interactions between CYP2E1 and miR-378 [25], and CYP2C8 and miR-103/miR-107 [26]. Hepatocyte nuclear factor 4α (HNF4α) is a transcription factor regulating endo/xenobiotics-metabolizing enzymes and transports [27]. Overexpression of miR-34 and miR-24 in HepG2 cells inhibiting HNF4α decreases CYP7A1 and CYP8B1 [28]. Cytochrome b5 expression is regulated by miR-223 in human liver [29]. MiRNAs maintaining metabolic homeostasis are shown in Figure 1.

## 3. Drug-Induced Liver Damage

Drug-induced liver injury (DILI) is an unusual but important adverse event of several drug classes. Its occurrence is estimated to be 14 to 19 cases per 100,000 persons, with 30% of cases showing jaundice as predominant symptom [30,31]. DILI is answerable for 3 to 5% of hospitalization for jaundice [32] and is the most common cause of acute liver failure in Western countries [33,34]. Numerous risk factors have been linked with its development [35]. Compared to children, adults seem to be at higher risk for DILI, although the pediatric population is prone to develop DILI with the use of valproic acid, anticonvulsants, antimicrobials, aspirin, or propylthiouracil [36,37]. A similar pattern has been observed in female patients, but they are not at higher risk of all-cause DILI [38,39,40]. HLA alleles, genetic polymorphisms of the CYP450 and other drug-processing enzymes have been recognized and linked to DILI. In this frame, CYP polymorphisms have been categorized in five metabolic phenotypes: (i) poor metabolizers, (ii) intermediate metabolizers, (iii) normal metabolizers, (iv) rapid metabolizers, and (v) ultra-rapid metabolizers [41]. Furthermore, drugs that are processed via CYP2C19 or CYP2C9 seem to be related to a greater risk of DILI than medicines processed by CYP2D6 or CYP3A. Drug–drug interactions can also yield hepatotoxicity. Alcohol use and malnutrition can also contribute to DILI, as observed in acetaminophen-mediated toxicity [42]. DILI is classically categorized as direct idiosyncratic [43] or indirect injury. Drugs produce direct hepatotoxicity with intrinsic liver toxicity. This injury is predictable, common, dose-dependent, and replicable in animal models. It occurs within one to five days after high therapeutic planned doses or overdose [44]. Agents trigger idiosyncratic hepatotoxicity with little or no intrinsic toxicity and only cause liver injury in rare cases [31,45]. It develops irrespective of drug dose, route, or administration duration and is related to a wide spectrum of clinical pattern presentation [37]. Indirect hepatotoxicity occurs when a drug promotes liver injury as part of its mechanism of action instead of a direct insult to the liver as in the case of checkpoint inhibitors [46] or monoclonal antibodies used for autoimmune diseases. Several pathophysiological mechanisms can contribute to DILI, among these are the direct impairment of hepatocellular structure (e.g., tetracycline, valproic acid, and tamoxifen-induced mitochondrial malfunction) and function, or the production of a metabolite that affects the structural and functional integrity of the liver. Additional mechanisms are the synthesis of a reactive drug metabolite that produces new antigenic entities through the binding with hepatic proteins. These drug–protein adducts are in turn targeted by hosts’ defenses triggering a systemic hypersensitivity response that damages the liver [47,48,49]. Understanding pathophysiological mechanisms enables idiosyncratic DILI to be categorized in two forms: immune-mediated (allergic] and non-immune-mediated (non-allergic) [50]. Typical of immune-mediated idiosyncratic DILI is a shorter latency (1–6 weeks] compared to non-immune-mediated reactions (1 month to 1 year] [51,52]. However, exceptions can occur with the appearance of immune reactions after a very long latency (e.g., nitrofurantoin) [53] and in some cases even after drug cessation (e.g., sulfonamides, erythromycin, and amoxicillin-clavulanate) [52]. Generally, DILI is due to a dose-dependent toxicity, however most drugs with hepatotoxic potential provoke idiosyncratic liver injury. Paracetamol is the most common cause of direct liver injury leading to hepatic acute injury or liver failure in supratherapeutic dosage [54]. This is because cytochrome-P_450-_-mediated acetaminophen hepatic metabolism produces a highly reactive (toxic) intermediate metabolite (i.e., N-acetyl-p-benzquinamides (NAPBQI) [55]. Normally, NAPBQI undergoes detoxification by glutathione conjugation in phase II reaction. However, during acetaminophen’s overdose, a high level of the toxic metabolite is produced, thus overwhelming the detoxification process. This process in turn leads to hepatocellular necrosis. Reports have demonstrated that liver injury mediated by this metabolite can be attenuated by the administration of acetylcysteine—a precursor of glutathione—acting as scavenger of the toxic metabolite [56]. Other important causes of direct DILI are methotrexate and amiodarone, of which chronic use induces liver fibrosis and cirrhosis in a dose- and time-dependent fashion. Acute hepatic necrosis can also be induced by high doses of aspirin, acetaminophen, niacin, and several antineoplastic agents [57,58,59]. Regarding idiosyncratic DILI, the causative drugs have diverse clinical phenotypes mimicking both acute and chronic liver disease. Acute hepatocellular hepatitis is frequently drug-induced idiosyncratic liver injury [31,37,60] and the agents usually involved are isoniazid, nitrofurantoin, and diclofenac [45,60,61,62]. Many drugs known as causative agents of acute hepatocellular injury can also lead to a chronic hepatocellular pattern [14,32] after months or years of exposure. Autoantibodies are frequently present and common causes of drug-induced, autoimmune-like chronic liver injury are nitrofurantoin, minocycline, hydralazine, methyldopa, statins, and fenofibrate [62,63]. Further clinical manifestations of idiosyncratic hepatotoxicity are the occurrence of cholestatic hepatitis or mixed hepatitis, both with favorable outcomes. Drug-induced cholestatic liver injury is often related to the administration of amoxicillin–clavulanate, cephalosporins, terbinafine, azathioprine, and temozolomide [63,64,65,66,67,68], while macrolide antibiotics and fluoroquinolone, sulfonamides, and phenytoin are common causes of drug-induced mixed hepatitis. Although the improvements in drug design and preclinical screening for toxic effects have led to drugs with better safety profiles and fewer hepatotoxic effects, many recently approved agents are still issue of concern—among these are the kinase and other targeted enzyme inhibitors. Most are antineoplastic drugs that induce transient increase in hepatic serum enzyme levels in a substantial proportion of patients, even though they rarely provoke jaundice or clinically apparent liver injury (e.g., imatinib, bortezomib, nilotinib, ribociclib, and pazopanib), [69]. Additionally, remarkable are MABs, which are frequently prescribed for cancer chemotherapy, however their therapeutic use has expanded, encompassing autoimmune diseases, migraines, hypercholesterolemia, and management after organ transplantation. Most monoclonal antibodies do not show hepatotoxicity except for those with immunomodulatory properties. Beside the classical pharmaceutical agents, one issue of concern is the hepatotoxic potential of herbal and dietary supplements (HDS). In a prospective study, HDS have been recognized as the most likely causative agent in 16% of cases of DILI [70]. However, the lack of a precise definition of DILI provoked by herbal and dietary supplements as well as the use of multiple HDS agents or the presence of a single implicated supplement of various compounds [70] mean that the attribution of herbal-induced liver injury to a single constituent is difficult. Remarkably, the clinical phenotype of hepatotoxicity related to herbal and dietary supplements is acute hepatocellular hepatitis, often severe and with a high rate of fulminant hepatic failure requiring liver transplantation [70]. Frequently involved components include green tea extracts (Camellia sinensis). Table 1 summarizes the drugs inducing DILI. The theoretical active molecular constituents are catechins, which at high doses cause liver injury in animal models. However, it must be pointed out that the amount of green tea used in the animal models were much higher than those in commercial supplements, thus the injury in humans was idiosyncratic and maybe immune-mediated [71].

## 4. miRNAs in DILI

**Figure 2:**
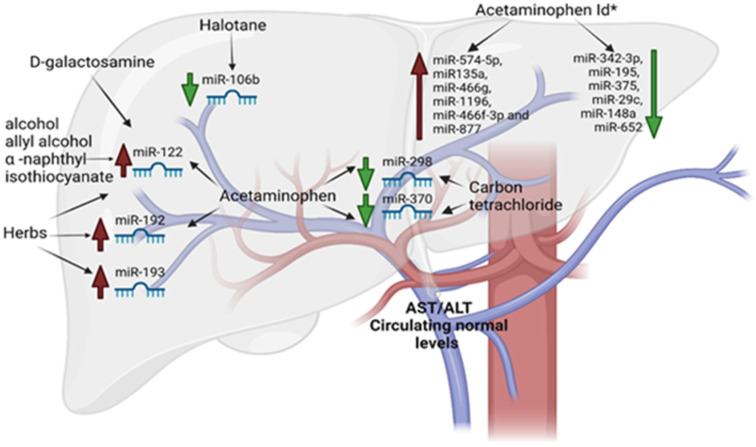
Early miRNAs released by liver in response to DILI.

The use of miRNAs as biomarkers from serum or plasma was first proposed in a DILI experimental model by pioneer study of Wang et al. They examined, in a well-established mouse model of acetaminophen-induced liver injury, the role of those molecules [72]. They demonstrated that the liver-enriched miR-122 and miR-192 were the top two miRNAs elevated in the blood in a dose- and time-exposure-dependent manner. The levels of these miRNAs preceded and then are parallel to serum ALT/AST levels. From this, several other independent groups provided additional data supporting the use of miRNAs as DILI biomarkers. In particular, the plasma levels of miR-122 were correlated with liver histopathology damage induced by D-galactosamine and alcohol [73]. This confirms, even in this case, that the plasma level fluctuations of miR-122 preceded largely the changes in transaminases blood level. Therefore, it was evident that this miR could be used for diagnosing and monitoring liver damage at early stages. Compared with mice, the susceptibility to acetaminophen-induced liver damage is lower in rats [74]. Therefore, rat models were used to examine acute liver damage (cholestasis or hepatocellular damage) and chronic liver damage (fibrosis, steatohepatitis and steatosis) [75]. Their results showed that miR-122 levels increased more quickly and intensely than levels of aminotransferases, reflecting the extent of liver damage. Outstandingly, the study also demonstrated that the expression profiles of plasma miRNAs differed according to the type of liver damage, suggesting that the miRNAs could be specific and sensitive biomarkers for various types of liver damage. An increase in levels of several oncogenic miRNAs, such as the 17–92 cluster, miR-106a, and miR-34, was detected in rat livers following exposure to tamoxifen, a potent hepatocarcinogen [75]. Furthermore, Starckx et al. demonstrated that the levels of miR-122 in rat plasma were significantly increased following administration of four well-characterized compounds associated with different types and mechanisms of liver toxicity to chemicals allyl alcohol and α-naphthyl isothiocyanate, and drugs acetaminophen and phenobarbital [76]. The changes in plasma miR-122 were detected significantly earlier than other conventional biomarkers, exhibiting a wide dynamic range. Thus, miR-122 is expected to be an early biomarker of DILI. Additionally, it was reported that the exposure to acetaminophen or carbon tetrachloride results in a decrease in miR-298 and miR-370, which are thought to regulate an oxidative-stress-related gene [77]. Furthermore, lethal doses of acetaminophen lead to miR-1196, miR135a, miR-466f-3p, miR-466g, miR-877, and miR-574-5p upregulation, and a marked downregulation of miR-195, miR-375, miR342-3p, miR-29c, miR-652, and miR-148a [78,79,80,81]. It was reported by Su et al. that miR-192, miR-193, and miR-122 have the potential to aid as specific, sensitive, and noninvasive biomarkers for the diagnosis of herb-induced liver damage [82]. The hepatotoxic drug halothane in patients with low expression of miR-106b caused the upregulation of signal transducer and activator of transcription 3 (STAT3) involved in its severe liver damage [83]. In Table 2 and Figure 2 the circulating microRNAs as early biomarkers for DILI are summarized.

Circulating miRNAs as biomarkers in human DILI showed that the levels of both miR-192 and miR-122 were considerably higher in patients who suffered acetaminophen-induced liver damage than in those who did not [83]. In humans, Bala et al. discovered that serum plasma levels of miR-155 and miR-122 were mainly related to the exosome-rich fraction in inflammatory and alcoholic liver damages (hepatitis), while in acetaminophen-induced liver damage, these miRNAs were present primarily in the soluble protein-rich fraction [74]. Of note, circulating levels of the liver-enriched miR-122, but not miR-192, were associated with serum transaminase levels and a decrease to baseline much earlier than serum transaminase, suggesting that miR-122 has a shorter circulatory half-life [80]. Subsequently, it was also demonstrated that miR-19b and miR-29c were upregulated after low-dose acetaminophen treatment without ALT alteration, suggesting that the expression profile of circulating miRNAs may be changed at a very early stage when liver damage is undetectable using the conventional biomarkers [82,83]. Paraquat is the most common toxic herbicide, and is widely used around the world. By associating paraquat-exposed human subjects with healthy donors, Ding et al. found that the serum levels of miR-122 were strongly increased and correlated well with the status of liver function [83], similarly to the mouse acetaminophen-induced liver damage model [80]. However, miR-192, also identified in the mouse study, was unpredictably decreased two-to-eight-fold in human hepatic samples. These studies provided convincing evidence that circulating miRNAs could be used as a human DILI biomarker.

## 5. Hepatitis B Virus: Genome of and Pathogenesis

The Hepatitis B virus (HBV) is the best-known member of the Hepadna virus family. Infection with HBV can cause chronic hepatitis, liver cirrhosis, and hepatocellular carcinoma (HCC) [83]. The HBV genome is a spherical particle of 42 nm in diameter, with a length of 3.2 kb. HBV is a member of the hepadnaviridae family, consisting of an outer shell or major protein (SHBs or HBs Ag: Hepatitis B Surface Antigen] enveloping a central core (core) with a diameter of 27 nm. The HBV genome is a relaxed, incomplete, circular double-stranded DNA molecule [84], formed by a longer chain with – or L (-) polarity of about 3200 nucleotides and by a shorter one with polarity + or S (S +) which has a length ranging from 1700 to 2800 nucleotides. HBV DNA sequencing shows four important gene sequences (open reading frame ORFs) placed on the negative polarity chain and named S, C, P, and X. The S gene encodes HBsAg, as mentioned above, and is preceded by two regions called pre-S2 and pre-S1 which encode two other surface proteins: the middle protein encoded by pre-S2 and S and the large protein encoded by pre-S1, pre-S2, and S [85]. Gene C encodes the nucleocapsid protein (HBc Ag) and is preceded by a region called the pre-core, which encodes a non-structural protein (HBe Ag). Region P codes for one polyprotein consisting of four functional sub-units, including reverse transcriptase and DNA polymerase. Region X encodes a protein of 17 kD which has transactivating activity [86,87], the HBV X protein (HBx). HBx is a highly conserved 154-amino acid protein that does not bind directly to the host DNA but activates various viral and cellular promoters [88]. The pathogenesis of liver damage from hepatitis B virus is still controversial and not fully accepted by all experienced researchers on this topic due to the lack of a valid experimental model. However, the data derived from studies carried out in the most accredited centers around the world orient on the fact that HBV is not a direct cytopathic virus, but that its pathogenic action and the succession of events that lead to healing or chronicization (both of the infection and of the disease) would be the consequence of a complex action of the immune system of the infected host. In this sense, the immune response against HBV is both humoral and cell-mediated. This arises from clinical, serological, and histological findings: high levels of viral replication (HBV DNA), HBe Ag, and the detection of the core antigen (HBc Ag) in nucleus of infected hepatocytes can coexist, in fact, with a histology of the completely normal liver and serological biochemistry [89,90]. In fact, the hypothesis that immune reactions (both humoral and cellular-mediated) are directed towards the viral antigens expressed on the plasma membrane of infected cells can play a decisive role in inducing necrosis of hepatocytes and, therefore, also the outcome of the disease [91,92,93]. An important role in this regard is also played by non-viral inactivation cytolytic by some inflammatory cytokines (Interferon-α2, IL-10, and TGF-β1) released from virus-activated mononuclear cells [94]. Neutralizing antibodies, which in cases with favorable evolution appear early after infection, are likely to be specific to antigenic determinants present on the viral envelope whose production is encoded from the pre-S1 and pre-S2 genomic regions. The identification and elimination of infected hepatocytes is up to T-cytotoxic lymphocytes [95]. Cytotoxic activity (HLA-I-restricted] depends on the recognition of some antigenic determinants present on the hepatocyte membrane during the phase viremic. They are likely to consist of peptide fragments of the core (HBc Ag and HBe Ag) and the viral coating [95]. The intracellular clearance of viruses occurs through direct contact of T-cytotoxic lymphocytes (HLA-I-restricted) with the infected cell. Cell necrosis follows the induction of apoptosis and the secretion of soluble cytokines that recall other cells’ effector immunity (macrophages and neutrophils) at the site of infection [95]. CD4 + lymphocytes (helper T lymphocytes) also play an important role: in patients, there is an important T antibody response helper (HLA-II restricted) to the different epitopes of core antigens (HBc Ag and HBe Ag) which is evident when HBs Ag disappears from the serum [95]. Furthermore, some data would suggest the predominant role of the lymphocyte response with TH1 cytochemical profile, processing IL-2, IFN-ϒ, and TNF-β) [93,94,95]. HBs Ag has a fair amount of antigenic variability and is precisely based on the latter, which was proposed to classify HBV into eight genotypes (A-H plus a genotype U Undetermined) and several subgenotypes. G and H genotypes are distributed mainly in Mexico. The F genotype is mainly distributed in Central and South America and, but genotypes A and D are also common in Brazil, Cuba, and Haiti. Genotype A predominates in Western Europe, while genotypes D and E in countries of the Mediterranean basin. The difference in the distribution of the genotype may be related to ethnicity and human migration [96]. The replication cycle of HBV is quite unique and still has some dark spots. Despite being a DNA virus, in fact, HBV replicates like a retrovirus: to duplicate its own genome it uses a pregenomic intermediate RNA as a template, on which the corresponding DNA is then copied by reverse transcription [97,98,99]. Once the virus penetrates inside the hepatocyte, it strips from the envelope. Then, the viral polymerase collects free nucleotides in the cell’s cytoplasm and initiates the completion of the short strand S, unpaired, of DNA starting from end 3 ‘, whose location is highly variable depending on the viral strain. Hence, the genome migrates to the nucleus where synthesis of the short S (+) chain is completed. This results in the conversion of incomplete double-stranded DNA into double-stranded DNA, complete circular, covalently closed (cccDNA), and supercoiling (supercoiled DNA). The transcription process takes place by a DNA-dependent RNA-polymerase host cell, cccDNA (serving as a template), and in multiple forms of RNA genomic is used as a model for reverse transcriptase; the same RNA acts also as a messenger for the synthesis of specific virus proteins (structural and otherwise). Then, there is the synthesis of the first DNA chain by reverse transcriptase associated with the virus core and after synthesis of the second DNA chain by copying the first.

## 6. miRNAs and Hepatitis B Virus

**Figure 3:**
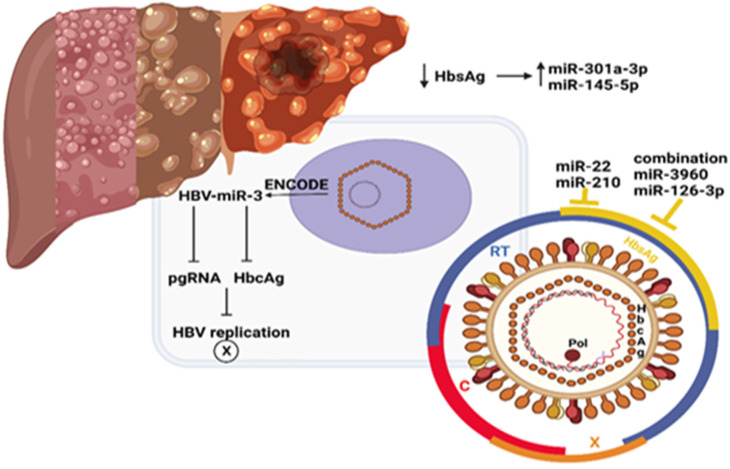
Antiviral HBV treatment influencing microRNAs.

Although HBV has infected 2 billion people worldwide [88], causes chronic hepatitis in comparison to healthy individuals, and HBV-infected patients have a 100-fold higher risk of developing hepatocellular carcinoma [100], to date, scarce data are available on this infection with microRNAs due to the lack of an experimental model. Only one HBV-encoded miRNA has been identified so far by deep sequencing of HBV-positive HCC tissue. The agreeing study identified and established the expression of HBV-miR-3. HBV-miR-3 downregulated HBV protein and HBV replication by reducing the expression of HBcAg, a positive regulator of HBV transcription, and pre-genomic RNA (pgRNA), which inhibits HBV replication overall [101]. However, no data are currently available on the role of HBV-miR-3 in HBV-associated liver damage as fibrosis/cirrhosis transition [102] (see Figure 3). It is important to remember here that genes encoding for miRNAs are transcribed by the RNA polymerase II/III into primary RNA transcripts (pri-miRNA), then processed by the Drosha ribonuclease to a hairpin loop structure of about 55–60 nucleotide (pre-miRNA) in nucleus. These are then exported to the cytoplasm and cleaved by a Dicer enzyme into mature double-stranded miRNA. No data are currently available on the role of HBV infection on liver microRNAs profile due to the lack of solid experimental models. In any case, there are evidence that plasma miRNA expression varies in response to antiviral treatment and it could be a promising tool for pharmacological treatment selection. Recently, higher levels of miR-301a-3p and miR-145-5p in patients with HBsAg loss were shown. Even a combination of miR-3960 and miR-126-3p were correlated with the clearance of HBsAg [102,103,104]. In patients receiving either interferon or nucleoside analogues, the signature of specific miRNAs and miR-B index was assessed, combining serum miR-99, miR-335, miR-192, miR-126, miR-122, and miR-320 for the prediction of a sustained virologic response, although little correlation was seen with miR-210, miR-22, and ALT or with HBsAg or HBeAg clearance [105,106,107,108,109].

## 7. Conclusions

The estimate, diagnosis, and management of DILI are very complex issues. The current biomarkers or methods to assess DILI include biochemical markers with poor sensitivity, stability, or specificity. In pre-clinical settings, circulating levels of liver-specific miR-122, the more abundant miR in the liver, can consistently and effectively distinguish intrahepatic from extrahepatic damage with higher sensitivity and specificity in pre-clinical settings. The convincing evidence that circulating miR-122 could be used as a human DILI biomarker comes from several studies in which it was evidenced that a strong increase in miR-122 correlated well with the status of liver function. In infancy, there is instead the possibility to have an early miRNA screening for hepatitis B virus infection. miRNAs hold yet unrealized potential as biomarkers for the early detection of hepatocellular carcinoma mediated by HBV.

## Figures and Tables

**Table 1:** Drugs inducing DILI.

Drugs	DILI
Checkpoint inhibitors, Immunomodulatory MABs	Indirect hepatotoxicity
Tetracycline, Valproic acid, Tamoxifene	Mitochondrial dysfunction
Nitrofurantoin, Sulfonamides, Erythromycin, Amoxicillin-clavulanate	Immune-mediated idiosyncratic DILI
Metotrexate, Amiodarone	Liver fibrosis, Cirrosis
Acetaminophen, Aspirin, Niacin, Antineoplastic agents	Acute hepatic necrosis
Isoniazid, Nitrofurantoin, Diclofenac	Acute hepatocellular hepatitis
Nitrofurantoin, Minocycline, Hydralazine, Methyldopa, Statins, Fenofibrate	Autoimmune-like chronic liver injury
Amoxicillin–clavulanate, Cephalosporins, Terbinafine, Azathioprine, Temozolomide	Cholestatic liver injury
Fluoroquinolone and Macrolide antibiotics, Phenytoin, Sulfonamides	Mixed hepatitis
Imatinib, Nilotinib, Bortezomib, Pazopanib, Ribociclib	Jaundice (rare)
Green tea extracts	Acute hepatocellular hepatitis

**Table 2:** Circulating miRNAs as early DILI biomarkers.

Drugs and Chemicals	microRNA Upregulated	microRNA Downregulated
D-galactosamine	miR-122	
Tamoxifen	miR-106a, 17–92 cluster, and miR-34	
Acetaminophen	miR-122 and miR-192	miR-298 and miR-370
Acetaminophen ld *	miR-1196, miR135a, miR-466f-3p, miR-466g, miR-877 and miR-574-5p	miR342-3p, miR-195, miR-375, miR-29c, miR-148a and miR-652
Alcohol	miR-122	
Allyl alcohol	miR-122	
α -naphthyl isothiocyanate	miR-122	
Carbon tetrachloride		miR-298 and miR-370
Halothane		miR-106b
Herbs	miR-193, miR-192, and miR-122	

* ld: lethal doses.

## Data Availability

Not applicable here.

## Abbreviations

ABCA1	ATP-binding cassette transporter
ABCG1	ATP Binding Cassette Subfamily G Member 1
AKT	AKT serine/threonine kinase 1
AMPK	AMP-activated protein kinase
Bmpr1a	bone morphogenetic protein receptor type 1A
CPT1α	Carnitine palmitoyltransferase I
CROT	Carnitine O-Octanoyltransferase
FOXA1	Forkhead box protein A1
G6PC	glucose-6-phosphatase
GSK	Glycogen Synthase Kinase
HAMP	Hepcidin Antimicrobial Peptide
HMGCR	3-Hydroxy-3-Methylglutaryl-CoA Reductase
HNF6	hepatocyte nuclear factor 6
HNFA4	hepatocyte Nuclear Factor 4 Alpha
LXRα	liver X receptor alpha
mTOR	mammalian target of rapamycin
MTT	microsomal triglyceride transfer protein
NPC1	NPC intracellular cholesterol transporter 1
NRF2	nuclear factor erythroid 2-related factor 2
PIK3	phosphatidylinositol-3-kinase
PTEN	Phosphatase and Tensin Homolog
SIRT1	Sirtuin 1
SIRT6	Sirtuin 6
SREBP2	Sterol regulatory element-binding protein 2
VLDL	Very Low-Density Lipoprotein
